# HSP90 Contributes to *chs3-2D*-Mediated Autoimmunity

**DOI:** 10.3389/fpls.2022.888449

**Published:** 2022-06-03

**Authors:** Junxing Lu, Wanwan Liang, Nanbing Zhang, Solveig van Wersch, Xin Li

**Affiliations:** ^1^College of Life Science, Chongqing Normal University, Chongqing, China; ^2^Michael Smith Laboratories, University of British Columbia, Vancouver, BC, Canada; ^3^Department of Botany, University of British Columbia, Vancouver, BC, Canada

**Keywords:** plant immunity, HSP90, CHILLING SENSITIVE 3, *SNIPER1*, sensor NLR, helper NLR, CHS3, *CSA1*

## Abstract

Plants employ multi-layered immune system to fight against pathogen infections. Different receptors are able to detect the invasion activities of pathogens, transduce signals to downstream components, and activate defense responses. Among those receptors, nucleotide-binding domain leucine-rich repeat containing proteins (NLRs) are the major intracellular ones. CHILLING SENSITIVE 3 (CHS3) is an Arabidopsis NLR with an additional Lin-11, Isl-1 and Mec-3 (LIM) domain at its C terminus. The gain-of-function mutant, *chs3-2D*, exhibiting severe dwarfism and constitutively activated defense responses, was selected as a genetic background in this study for a forward genetic screen. A mutant allele of *hsp90.2* was isolated as a partial suppressor of *chs3-2D*, suggesting that HSP90 is required for CHS3-mediated defense signaling. In addition, HSP90 is also required for the autoimmunity of the *Dominant Negative (DN)-SNIPER1* and gain-of-function *ADR1-L2 D484V* transgenic lines, suggesting a broad role for HSP90 in NLR-mediated defense. Overall, our work indicates a larger contribution of HSP90 not only at the sensor, but also the helper NLR levels.

## Introduction

In nature, plants are challenged by various microbial pathogens, including viruses, bacteria, fungi, and oomycetes. They have evolved a complicated immune system in order to combat infections. Both pattern-triggered immunity (PTI) and effector triggered immunity (ETI) which relies on intracellular receptors nucleotide-binding domain leucine-rich repeat containing proteins (NLRs), can be activated during defense (Jones and Dangl, 2006). In some cases, ETI responses require two types of NLR proteins. One acts as the effector-sensing or sensor NLR. The other serves roles in signaling but may not participate in effector recognition. The latter group is termed helper NLRs (hNLRs) (Bonardi et al., 2011). ACTIVATED DISEASE RESISTANCE 1 (ADR1), N REQUIREMENT GENE 1 (NRG1) and NB-LRR REQUIRED FOR HYPERSENSITIVE RESPONSE-ASSOCIATED CELL DEATH1 (NRC1) are the three characterized hNLR families. Both ADR1 and NRG1 are CNLs (coiled coil type NLR) with N terminal regions similar to the Resistance to powdery mildew 8 (RPW8) domain. Different Toll/Interleukin-1-receptor (TIR)-type sensor NLRs (TNLs) seem to differentially employ ADR1s and NRG1s to transduce downstream defense signals (Wu et al., 2019).

NLRs are able to recognize effectors directly or indirectly. CHILLING SENSITIVE 3 (CHS3) is one of the TNLs with an atypical C-terminal Lin11, Isl-1 and Mec-3 (LIM) domain. The single amino acid substitution C1340Y close to the LIM domain leads to the gain-of-function *chs3-2D* mutant with autoimmune phenotypes (Xu et al., 2015). We previously conducted a *chs3-2D* forward genetic screen by using the chemical mutagen ETHYL METHANE SULFONATE (EMS) and found that the autoimmunity of *chs3-2D* fully requires the adjacent TNL gene *CONSTITUTIVE SHADE-AVOIDANCE 1* (*CSA1*), but marginally relies on PAD4, which is a positive regulator downstream of many TNLs (Xu et al., 2015; Lapin et al., 2022).

*SNIPER1* (*snc1*-influencing plant E3 ligase reverse genetic screen, 1) and its homolog *SNIPER2* globally control the protein levels of sensor NLRs, but not hNLRs. Upon *SNIPER1* overexpression, sensor NLRs’ levels were reduced and resulted in enhanced disease susceptibility to pathogens. In contrast, more sNLR accumulation and enhanced disease resistance were observed when the dominant-negative (DN) form of *SNIPER1* was expressed (Wu et al., 2020). However, SNIPER1 has no effect on the autoimmunity mediated by the gain-of-function helper NLRs ADR1-L2 D484V or NRG1A D485V (Wu et al., 2020). The direct E3-substrate relationship between SNIPER1 and the sNLRs seems to be through the well conserved NB domain, which exists in all typical plant sensor NLRs (Wu et al., 2020).

Here, we describe an independent suppressor screen in the *chs3-2D* background, which was carried out to identify new signaling components of CHS3-mediated defense. Multiple mutants that can suppress the *chs3-2D* autoimmune phenotypes were identified and characterized. Using mapping-by-sequencing, we found mutations in a number of genes, including novel alleles of known regulators, such as *IBR5* (INDOLE-3-BUTYRIC ACID RESPONSE 5) and *CSA1* (Liu et al., 2015; Xu et al., 2015). In addition to the known regulators, our study revealed that the autoimmunity of *chs3-2D* is also partially suppressed by mutation in *HSP90.2*.

## Materials and Methods

### Plant Growth

Seeds were sterilized by soaking them in a solution of 15% bleach and 0.1% Tween 20 followed by rinsing twice with sterile water. For soil-grown plants, seeds were stratified at 4°C for two days, sown onto sterile soil and transferred to plant growth rooms with either room temperature (23°C, 16 h light/8 h dark; ∼50% relative humidity) or high temperature (28°C, 16 h light/8 h dark; ∼50% relative humidity) conditions as specified in figure legends. Plate-grown plants were grown on half strength Murashige and Skoog (1/2 MS) medium at 22°C and exposed to a 16 h light and 8 h dark regime.

### Ethyl Methane Sulfonate Mutagenesis, Mutant Screens, and Next Generation Sequencing

The *chs3-2D* suppressor screen was carried out as previously described using an EMS-mutagenized *chs3-2D* population (Li and Zhang, 2016). 100 mg (∼5000) *chs3-2D* seeds were soaked in a solution of 0.1 M sodium phosphate (pH 5), 5% dimethyl sulfoxide, and 0.25% EMS for 16 h with constant shaking. After incubation, the seeds were washed twice in 100 mM sodium thiosulphate for 15 min and three times in distilled water for 15 min. The mutagenized *chs3-2D* seeds were planted on 1/2 MS plate for 10 days. Then ∼4000 seedlings were transplanted on soil and grown at 28°C as the dwarfism and lethality of *chs3-2D* can be suppressed at 28°C. M2 seeds from 25 M1 plants were harvested in each pool. For screening, ∼500 M2 seeds from each pool were planted. Mutants were identified in M2 by selecting plants completely or partially reverted to WT morphology in the extremely dwarfed mutant background (Bi et al., 2011). Mutants were backcrossed with *chs3-2D*; F1s are heterozygous for the mutated gene, while 25% of F2s are homozygotes if the suppression is caused by single nuclear gene mutation. We followed homozygous F2s to F3 generation to confirm their phenotypes, and pooled F3 plants were subjected to the next generation sequencing (NGS) analysis.

For next generation sequencing, total genomic DNA was extracted from 24-day-old candidate suppressors using the CTAB method (Li et al., 2001) followed by DNA purification using Qiagen plant DNA extraction kit (Qiagen, Germany). The library preparation and Illumina sequencing were carried out by the BGI (Beijing Genomic Institute, China).

### Construction of Plasmids and *Arabidopsis* Transformation

The genomic fragment of *At5G56030* containing its native promoter was PCR-amplified from wildtype (WT) Col-0 genomic DNA. The fragment was then digested and cloned into the vector *pCambia1305* to generate *HSP90.2:HSP90.2*. For *Arabidopsis* transformation, the above constructed binary vectors were transformed into *Agrobacterium tumefaciens GV3101* (pMP90) by electroporation and subsequently transformed into *Arabidopsis* plants by the floral dip method (Clough and Bent, 1998). Transgenic plants were screened on soil by spraying 100 mg/L BASTA four times with a two-day interval between each treatment.

### Identifying Double Mutants

The mutants *hsp90.2* G122S, *hsp90.3* S100F (Huang et al., 2014) and *sgt1b* (Austin et al., 2002) were used to generate double mutants with *DN-SNIPER1* (Wu et al., 2020) and *ADR1-L2 D484V* (Roberts et al., 2013). To identify homozygous double mutants, F1s were planted on BASTA selection plates and genotyped for heterozygous backgrounds. F1s were individually harvested, and F2s were planted on BASTA selection plates to select for *ADR1-L2 D484V* or *DN-SNIPER1* transgenes. Only plants that showed no segregation and were homozygous for the transgenes in F3 were genotyped for verification of homozygosity of the mutated *HSP90* chaperone gene.

### Oomycete Infection Assay

Three-week-old seedlings were spray-inoculated with *Hyaloperonospora arabidopsidis* (*H.a*.) Noco2 at a concentration of 1 × 10^5^ spores per ml. The oomycete was allowed to propagate in a humid growth chamber (12 h light/12 h dark, 18°C) for 7 days before the number of spores on the plant surface was quantified. The 12 plants from each genotype were divided into groups of three and placed in 1 ml of ddH2O in 15 ml test tubes (4 plants per tube). Spores were suspended in solution by vortexing and counted using a hemocytometer. Three independent replicates were performed.

### Statistical Analysis

The GraphPad Prism 9.0 software (GraphPad Software, Inc.) and Microsoft Excel were used for the statistical analyses in this study.

## Results

### Identification and Characterization of Full and Partial Suppressors of *chs3-2D*

The *chs3-2D* autoimmune mutant exhibits severe dwarfism (Bi et al., 2011). In order to search for components required for CHS3-mediated defense response, a suppressor screen was conducted. Suppression of stunted growth was used as a criterion during the primary screen. The genetic background of each mutant was verified by genotyping the *chs3-2D* locus. Then, mutants exhibiting morphological suppression of *chs3-2D*-associated phenotypes were subjected to a secondary screen, where resistance to the virulent oomycete strain *Hyaloperonospora arabidopsidis* (*H.a.*) Noco2 was examined. Mutants that displayed enhanced susceptibility to *H.a.* Noco2 as compared to *chs3-2D* were selected for further characterization.

**FIGURE 1:**
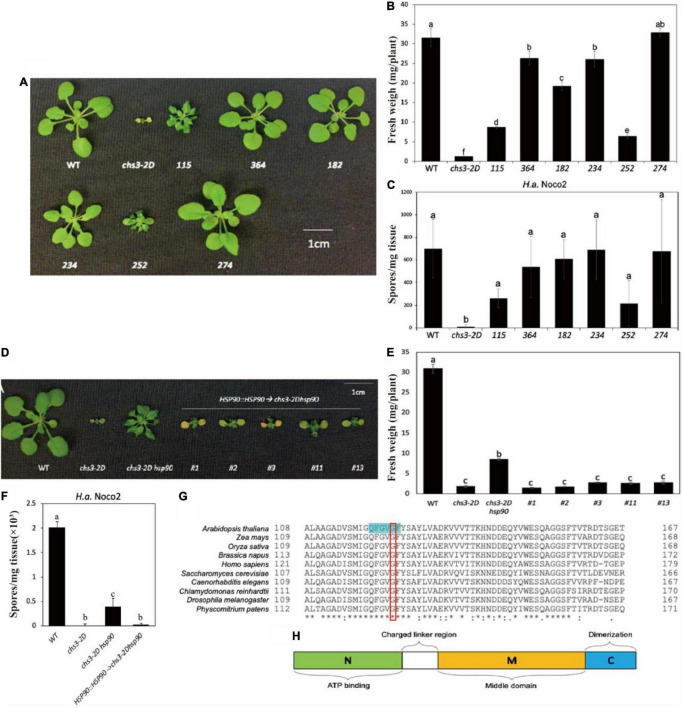
Identification and characterization of *chs3-2D* suppressors. **(A)** Morphology of 3-week-old soil-grown plants of *chs3-2D* suppressors at 18 °C. **(B)** Weights of 3-week-old soil-grown plants of *chs3-2D* suppressors at 18 °C. **(C)** Quantification of *H.a.* Noco2 sporulation on the leaf surface of *chs3-2D* suppressors. **(D)** Morphology of 3-week-old soil-grown plants of the mutant *chs3-2D hsp90.2* complementary transgenic lines at 18 °C. **(E)** Weights of 3-week-old soil-grown plants of the mutant *chs3-2D hsp90.2* complementary transgenic lines at 18 °C. **(F)** Quantification of *H.a.* Noco2 conidia growth on the leaf surface of the mutant *chs3-2D hsp90.2* complementary transgenic lines. **(G)** Amino acid alignments of selected HSP90 homologs from eukaryotic organisms. The amino acids highlighted in green are the nucleotide binding site. The mutated amino acid is labeled with a red box. For panel **(G)**, List of the organisms included: mouse-ear cress *Arabidopsis thaliana* HSP90.2, maize *Zea mays* (C3UZ63), rice *Oryza sativa* (Q0J4P2), rapeseed *Brassica napus* (A0A078GRJ1), human *Homo sapiens* (CAA33259.1), yeast *Saccharomyces cerevisiae* (AJS65103.1), nematode *Caenorhabditis elegans* (NP_506626.1), alga *Chlamydomonas reinhardtii* (XP_001695264.1), fruit fly *Drosophila melanogaster* (NP_001261362.1), and moss *Physcomitrium patens* (XP_024385281.1). Protein sequences were aligned using CLUSTAL. **(H)** Schematic diagram of the HSP90 domains. Hsp90 is comprised of three domains: an N-terminal ATP-binding domain (N, green) that may contain a peptide binding element; a middle domain (M, yellow) that interacts with client proteins and also contains a loop that catalyzes ATP hydrolysis; and a C-terminal dimerization domain (C, blue). A charged region exists between the N and M domains (linker region, white). Data information: For panels **(C,F)**, Two-week-old soil-grown seedlings were sprayed with a spore suspension of *H.a.* Noco2 at a concentration of 100,000 spores/ml of water. The plants were then covered and incubated for seven days in a high humidity growth chamber. Spores were counted in water suspension using a hemocytometer (bars represent means of n replicates ± SD, *n* = 3 with 4 plants each). One-way ANOVA followed by Tukey’s *post hoc* test were performed for panels **(A–F)**. Statistical significance is indicated by different letters (*P* < 0.05). Error bars represent mean SD (*n* = 5).

From the screen, six independent *chs3-2D* suppressor lines were isolated. As shown in Figures 1A,B, all six *chs3-2D* suppressors can either partially or fully suppress the morphology of *chs3-2D*. Consistent with the morphological suppression, all of them lost the constitutive resistance response of *chs3-2D* against *H.a.* Noco2 (Figure 1C). Taken together, these data suggest that all six mutants contain mutations suppressing the autoimmune phenotypes of *chs3-2D.*

These six independent *chs3-2D* suppression lines were backcrossed with *chs3-2D* separately. Mapping-by-sequencing was used on the co-segregating homozygous suppressor plants in F2 and F3, and the NGS data can be found at NCBI GenBank (PRJNA820297). Table 1 summarizes the candidate causal mutations identified in these mutants. Most of them contain mutations in known genes where loss-of-function mutations would suppress *chs3-2D* (Liu et al., 2015; Xu et al., 2015). Mutant *252* carried a point mutation in the fifth exon of *CHS3* (At5g17890), resulting in a P965 to S965 substitution. In addition, the complete suppressor *234-1* had a G to A splice site mutation in the first intron of *Indole-3-Butyric Acid Response 5* (*IBR5*, At2G04550), where the encoded protein is known to physically interact with CHS3 and is required for its defense responses (Liu et al., 2015). Lastly, *364*, *274-1*, and *182-1* carried mutations in *CSA1* (At5G17880) (Xu et al., 2015). One *csa1* allele had a point mutation in the third exon, leading to amino acid change from K862 to E862. The other two alleles contained mutations in the second and first exons, respectively, which result in early stop codon in premature protein (Table 1).

**TABLE 1:** Mutations in the six *chs3-2D* suppressor mutants.

Mutants	Gene code	Gene name	Mutations position	Amino acid changes
115	AT5G56030	HSP90.2	3rd exon	G122S
364	AT5G17880	CAS1	3rd exon	K862E
274-1	AT5G17880	CAS1	2nd exon	W205STOP
182-1	AT5G17880	CAS1	1st exon	W85STOP
234-1	AT2G04550	IBR5	1st intron	No amino acid change*
252	AT5G17890	CHS3	5th exon	P965S

Mutations were identified by next generation sequencing. The symbol (*) indicates splice site mutation from nucleotide G to A.

The *chs3-2D* partial suppressor, mutant *115*, carried a mutation in the third exon of *HSP90.2* (At5G56030), leading to an amino acid change from G122 to S122 (Table 1), suggesting that *HSP90.2* may positively regulate CHS3-mediated defense responses. To confirm that the partial suppression phenotype of the mutant *115* is due to the mutation in *HSP90.2*, a wild-type copy of *HSP90.2* driven by its native promoter was transformed in *115* mutant plants. The *115* mutant lines harboring the *HSP90.2* transgene reverted to a smaller size (Figure 1D) and lower weight (Figure 1E), and showed enhanced resistance against *H.a* Noco2 (Figure 1F) compared with the *115* mutant plants, comparable to *chs3-2D*. Therefore *HSP90.2* can fully complement the partial suppression phenotypes of mutant *115*, suggesting that the mutation in *HSP90.2* is responsible for partial suppression of the *chs3-2D* in *115*.

BLAST analysis revealed that the mutated amino acid G122 in HSP90.2 is conserved among HSP90 homologs from various eukaryotic organisms (Figure 1G). Furthermore, G122 is located at the central nucleotide binding site (Figure 1H; Xu et al., 2012). Thus, the mutation from G122 to S122 might cause a loss of HSP90.2 function. In Arabidopsis, HSP90.2 is redundant with HSP90.1/3/4. When we compared the current *hsp90.2* allele with other previously reported *hsp90.1/2/3/4* alleles (Table 2), this *hsp90.2* G122S mutation seems to be a novel mutant allele of *HSP90.2*.

**TABLE 2:** Reported *hsp90* mutant alleles in *Arabidopsis thaliana.*

Mutant name	Mutation position	Mutant phenotypes	Sources
hsp90.1-1; hsp90-1	T-DNA (SALK_007614)	Compromised RPS2-dependent resistance; Exhibited high H_2_O_2_ level	Takahashi et al., 2003; Toumi et al., 2019
hsp90.1-2	T-DNA (SALK_075596)	Compromised RPS2-dependent resistance	Takahashi et al., 2003
*hsp90.2-1*	G95E	Loss of recognition of *avrRpm1*	Hubert et al., 2003, 2009
*hsp90.2-2*; *hsp90.2-4*; *such2-1*	S100F	Loss of recognition of *avrRpm1*; suppressed the chilling sensitivity of *rpp4-1d*	Hubert et al., 2003, 2009; Bao et al., 2014
*hsp90.2-3*	D80N	Loss of recognition of *avrRpm1*	Hubert et al., 2003, 2009
*hsp90.2-5*	T-DNA (SALK_058553)	No alteration of RPM1-mediated resistance	Hubert et al., 2003
*hsp90.2-6*	A42T	Loss of recognition of *avrRpm1*	Hubert et al., 2009
*hsp90.2-7*	A11T	Suppressed *rar1* phenotypes; restoration of NB-LRR function and accumulation in a *rar1* mutant	Hubert et al., 2009
*hsp90.2-8*	R377C	Suppressed *rar1* phenotypes; restoration of NB-LRR function and accumulation in a *rar1* mutant	Hubert et al., 2009
*hsp90.2-10*	T-DNA	Loss-of-function mutant, cannot rescue the phenotype of *rpp4-1d* under chilling stress	Bao et al., 2014
*hsp90.2-11*; *muse12*	R33H and D41N	Enhanced the *snc1* phenotypes; increased of SNC1 accumulation	Huang et al., 2014
*hsp90.2*	G122S	Suppressed *chs3-2D*, gain-of-function helper NLR (*ADR1-L2 D484V*) and overaccumulation of sensor NLRs (*DN-SNIPER1*)	This study
*hsp90.3-1*; *such1-1*; *muse10*	S100F	Suppressed the chilling sensitivity of *rpp4-1d*; compromised RPM1-, RPS4- and RPP4-mediated mediated pathogen resistance; enhanced the *snc1* phenotypes; heightened accumulation of SNC1, RPS2 and RPS4	Bao et al., 2014; Huang et al., 2014
*hsp90.3-2*; *such1-2*	G124S	Suppressed the chilling sensitivity of *rpp4-1d*; compromised RPM1-, RPS4- and RPP4-mediated pathogen resistance	Bao et al., 2014
*hsp90.3-3*	T-DNA	Loss-of-function mutant, cannot rescue the phenotype of *rpp4-1d* under chilling stress	Bao et al., 2014
*hsp90.3-4*	T-DNA (SALK_013240)	Enhanced the *snc1* phenotypes, increased of SNC1 accumulation	Huang et al., 2014
*hsp90-4*	T-DNA (SALK_084059)	Exhibited high H_2_O_2_ level	Toumi et al., 2019

### Chaperones HSP90 and SGT1b Contribute to the Autoimmunity Caused by Overaccumulation of Sensor NLRs

**FIGURE 2:**
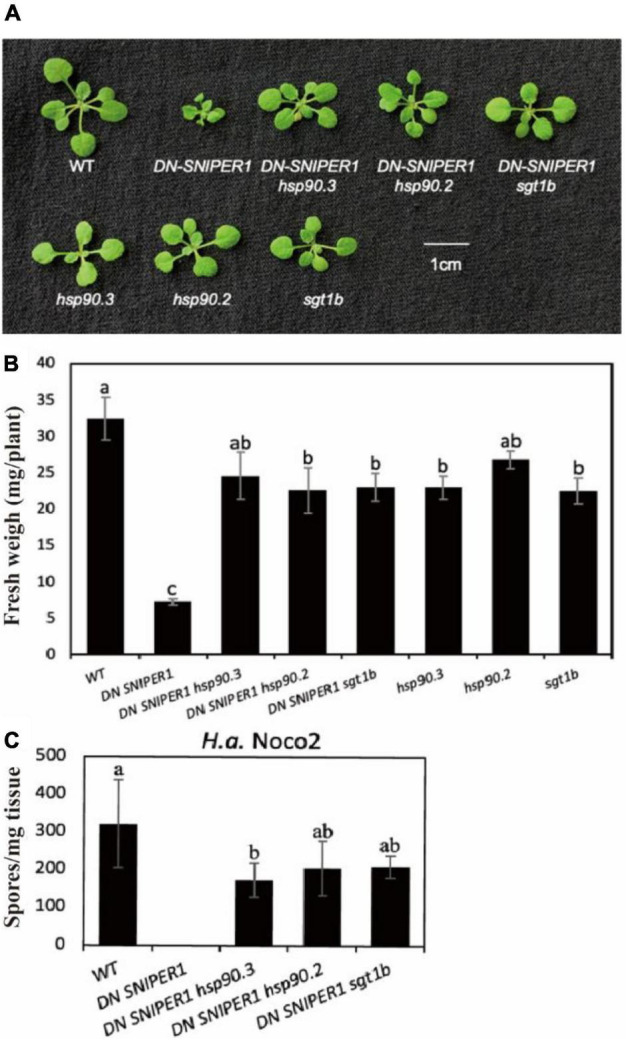
Chaperone protein HSP90s and SGT1b contribute to the autoimmunity caused by overaccumulation of sensor NLRs. **(A)** Morphology of 3-week-old soil-grown plants of *DN-SNIPER1 hsp90*, *DN-SNIPER1 sgt1b* double mutant at 23 °C. **(B)** Weights of 3-week-old soil-grown plants of *DN-SNIPER1 hsp90*, *DN-SNIPER1 sgt1b* double mutant at 23 °C. **(C)** Quantification of *H.a.* Noco2 conidia growth on the leaf surface of *DN-SNIPER1 hsp90*, *DN-SNIPER1 sgt1b* double mutant. Data information: For panel **C**, the experimental procedure was carried out as described in Figure 1. One-way ANOVA followed by Tukey’s *post hoc* test were performed for panels **(B,C)**. Statistical significance is indicated by different letters (*P* < 0.05). Error bars represent mean SD (*n* = 5).

As HSP90 and suppressor of the G2 allele of skp1 (SGT1) are known chaperons for NLRs, and loss-of-function mutations in *SGT1b* are known to suppress *chs3-1*, another autoimmune allele of *CHS3* (Kadota et al., 2010; Yang et al., 2010), we asked how widely HSP90 and SGT1b contribute to sensor NLRs. From our previous study, SNIPER1/2 can target a large range of sensor NLRs for ubiquitination and degradation. Overexpression of *SNIPER1* results in enhanced disease susceptibility, while a dominant-negative form of *SNIPER1* (*DN SNIPER1*) leads to more sensor NLRs accumulation and enhanced disease resistance (Wu et al., 2020). To address whether chaperone proteins HSP90 and SGT1b can regulate the autoimmunity caused by overaccumulation of sensor NLRs, we crossed *DN-SNIPER1* with *hsp90* or *sgt1b*. As shown in Figure 2, the dwarfism and enhanced resistance of *DN SNIPER1* against *H.a* Noco2 were largely suppressed by *hsp90.2*, *hsp90.3*, or *sgt1b*, indicating that chaperone proteins HSP90s and SGT1b are generally required by sensor NLRs.

### Mutation in *HSP90* or *SGT1b* Partially Suppresses Gain-of-Function Helper NLR Autoimmune Phenotypes

**FIGURE 3:**
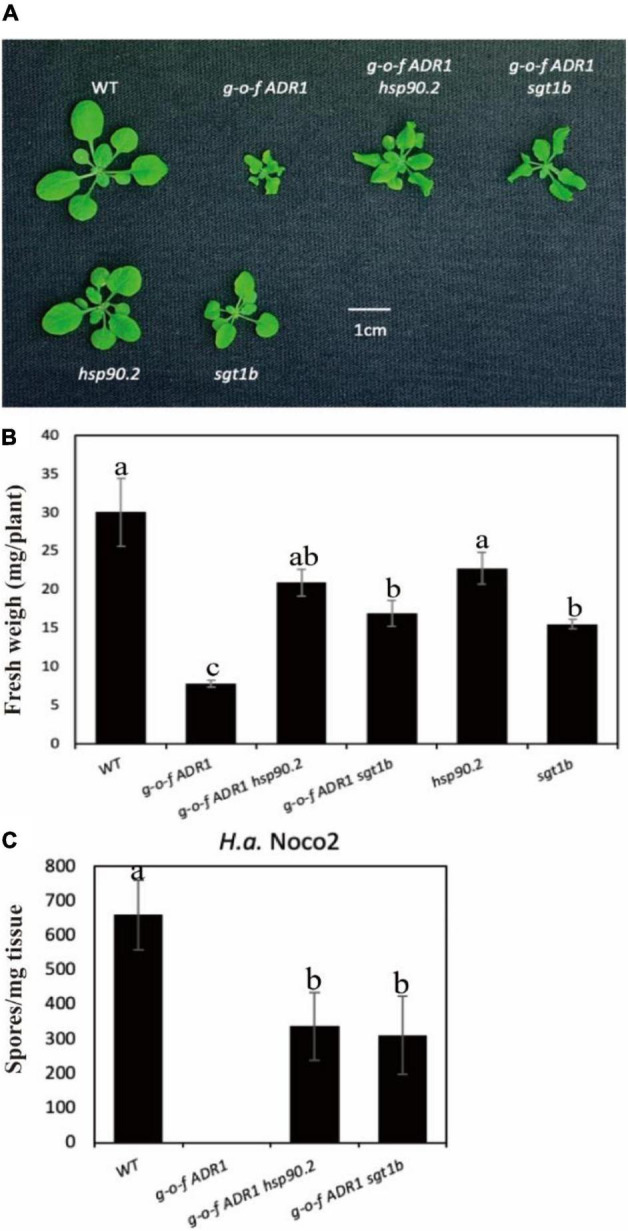
Mutations at HSP90s or SGT1b partially suppress the autoimmunity of *ADR1-L2 D484V*. **(A)** Morphology of 3-week-old soil-grown plants of *ADR1-L2 D484V hsp90, ADR1-L2 D484V sgt1b* double mutant at 23 °C. **(B)** Weights of 3-week-old soil-grown plants of *ADR1-L2 D484V hsp90, ADR1-L2 D484V sgt1b* double mutant at 23 °C. **(C)** Quantification of *H.a.* Noco2 conidia growth on leaf surface of *ADR1-L2 D484V hsp90, ADR1-L2 D484V sgt1b* double mutant. The experiment was carried out as described in Figure 1. One-way ANOVA followed by Tukey’s *post hoc* test were performed for panels **(B,C)**. Statistical significance is indicated by different letters (*P* < 0.05). Error bars represent mean SD (*n* = 5).

Helper NLRs serve functional roles downstream of sensor NLRs (Jubic et al., 2019; van Wersch et al., 2020). To test whether activation of helper NLRs also require chaperone/co-chaperone proteins HSP90 and SGT1b, we crossed a gain-of-function (g-o-f) variant ADR1 (*ADR1-L2 D484V*) (Roberts et al., 2013) with chaperone mutants *hsp90.2* or *sgt1b* (Figure 3). The double mutants *ADR1-L2 D484V hsp90* and *ADR1-L2 D484V sgt1b* showed partially suppressed morphological phenotypes (Figures 3A,B), which are consistent with the oomycete infection results (Figure 3C). This suggests the constitutive activation of defense responses in *ADR1-L2 D484V* also relies on chaperones HSP90 and SGT1b, as both *sgt1b* and *hsp90.2* can largely suppress the autoimmune phenotype of *ADR1-L2 D484V* plants. Taken together, general chaperone activities are not only required for sensor NLRs, but also helper NLRs.

## Discussion

The chaperones are a large and diverse group of unrelated proteins that control both non-covalent folding and post-translational maintenance of proteins in the cells (Liberek et al., 2008). They participate in diverse biological and cellular processes such as growth, development, disease resistance, and signal transduction (Kadota and Shirasu, 2012). Misfolded or misused sensors are threats to the cell and must be immediately inactivated and discarded to avoid inappropriate activation of downstream pathways. Therefore, chaperones are crucial in maintenance of NLR-type sensors (Shirasu, 2009). Interestingly, a point mutation allele of *hsp90.2* was identified from the *chs3-2D* suppressor screen. Our results show that this new allele of *hsp90.2* partially suppressed the autoimmune phenotypes of *chs3-2D* mutant plants (Figure 1), suggesting that *HSP90* contributes to CHS3-mediated defense responses. Combined with previous report where *sgt1b* and *rar1* were found to fully suppress *chs3-1* (Yang et al., 2010), HSP90, required for Mla12 resistance (RAR1) and SGT1 seem to be all serving chaperon roles for CHS3.

HSP90 chaperone proteins are highly conserved and abundant in diverse organisms, from bacteria to plants (e.g., moss, alga, rice, and maize) and mammals. In eukaryotes, HSP90 is involved in the assembly, stabilization and maturation of key signaling proteins, including protein kinases and hormone receptors (Kadota and Shirasu, 2012). It contains an N-terminal ATP-binding domain (ND), a middle domain (MD) for binding substrate proteins, and a C-terminal constitutive dimerization domain (CD) (Figure 1G). Plants without functional HSP90s may exhibit increased susceptibility to pathogens and reduced R protein mediated immunity and lower accumulation of immune receptors, suggesting that these chaperones are critical for NLR protein complex assembly, stability, and/or activation (Shirasu, 2009), which is consistent with our observation that a mutation in HSP90 can suppress the autoimmunity caused by a gain-of-function sensor NLR CHS3-2D (Figure 2). Such an observation is also consistent with previous studies suggesting that HSP90 chaperones are broadly involved in serving different sensor NLR client proteins, including RPM1, RPS5, Rx, MLA1, MLA6, and RPS2 (Hubert et al., 2003; Lu et al., 2003; Bieri et al., 2004; Holt et al., 2005; Kadota and Shirasu, 2012). Although we did not test the interactions between CHS3 or ADR1s with HSP90, we would expect that as NLRs, they likely also use the same chaperones for complex assembly during resistosome formation and defense activation.

In *Arabidopsis*, there are four cytosolic *HSP90* genes (HSP90.1-4), all locateed to the same region of chromosome 5 (Donato and Geisler, 2019; Krishna and Gloor, 2001). There is high degree of sequence homology among these paralogs, suggest functional redundancy (Queitsch et al., 2002; Hubert et al., 2003). Although the *Arabidopsis* HSP90 paralogs are highly related, they exhibit different expression profiles (Prasinos et al., 2005). Moreover, previous studies shown that different point mutations in HSP90s can cause drastically different or even opposite biological consequences (Supplementary Table 1 in Hubert et al., 2009; Table 2), supporting uneven functional redundancy among the family members. Our *hsp90.2* allele suppresses *chs3-2D*, gain-of-function helper NLR (*ADR1-L2 D484V*) and overaccumulation of sensor NLRs (*DN-SNIPER1*), indicating that HSP90 contributes broadly to chaperone both sensor and helper NLRs. As both sensor and helper NLRs require oligomerization for activation, HSP90, together with SGT1 and likely RAR1, probably assist in the assembly of resistosomes during NLR oligomerization and defense activation. However, it should be kept in mind that chaperones can not only assist with NLR receptor complex assembly during activation, they can also aid in other protein complexes’ organization during NLR degradation. For example, *hsp90.3* mutation affects the SCF^*CPR*1^ complex formation, leading to increased SNC1 levels and autoimmunity (Huang et al., 2014). Therefore, sometimes the phenotypes of an *hsp90* allele can be a combinatory consequence from both positive and negative effects of the HSP90 in different complexes, which may cause confusion in data interpretation.

The dimerized HSP90 usually works in association with co-chaperone proteins that modulate its ATPase activity or recruit substrates (Hoter et al., 2018). Studies in plants revealed that HSP90 and its co-chaperones, SGT1 and RAR1, are major stabilizing factors for NLR proteins (Kadota et al., 2010). HSP90 interacts with the cysteine-and histidine-rich zinc-binding domain (CHORD) of RAR1 as well as the CHORD domain and SGT1 motif of SGT1 (Boter et al., 2007). The Arabidopsis *SGT1* (*AtSGT1b*) gene was identified by loss of *Hyaloperonospora arabidopsidis* resistance in the *sgt1b* mutant that would otherwise be provided by RPP5 or RPP7 (Austin et al., 2002; Tor et al., 2002), indicating that SGT1 also plays an important role in NLR activation in plants. In plants, SGT1 is needed for the maintenance of steady-state levels of NLRs including Rx and N (Azevedo et al., 2006; Mestre and Baulcombe, 2006; Boter et al., 2007). Similar to HSP90, SGT1 not only positively regulates plant NLR activity such as CHS3, but also performs a negative role in controlling the turnover of NLRs such as RPM1, RPS5, and SNC1 (SUPPRESSOR OF NPR1, CONSTITUTIVE 1), as these NLR proteins accumulate to higher levels in *sgt1b* mutant plants (Holt et al., 2005; Li et al., 2010). The contribution of HSP90 and SGT1 in NLR turnover control can be explained by their contributions to the Skp1-Cullin-F-box (SCF) E3 ligase complex assembly for NLR ubiquitination and degradation (Huang et al., 2014; Kapos et al., 2019). The multi-functionality of SGT1 and HSP90 suggests a diverse client base involving different protein complexes assembled during immune responses (Huang et al., 2014). Furthermore, SGT1 is also needed for immune responses mediated by non-NLR-type sensors such as Cf4, Cf9, or RPW8 (Shirasu, 2009). Lastly, as mutations in either SGT1 or HSP90 can suppress autoactivation of helper NLR (Figure 3), both chaperones contribute not only at sensor NLR, but also helper NLR levels. Such broad roles should be taken into consideration for future biochemical studies of these chaperones in NLR biology.

## Data Availability Statement

The original contributions presented in the study are publicly available. This data can be found here: https://www.ncbi.nlm.nih.gov/, PRJNA820297.

## Author Contributions

JL, WL, NZ, and SW carried out the experiments. JL, NZ, and XL wrote the manuscript with inputs from all authors. XL conceived the original idea and supervised the project.

## Conflict of Interest

The authors declare that the research was conducted in the absence of any commercial or financial relationships that could be construed as a potential conflict of interest.

## Publisher’s Note

All claims expressed in this article are solely those of the authors and do not necessarily represent those of their affiliated organizations, or those of the publisher, the editors and the reviewers. Any product that may be evaluated in this article, or claim that may be made by its manufacturer, is not guaranteed or endorsed by the publisher.
